# Exploring Characteristics and Systemic Health Links in Myopic Australian Adults

**DOI:** 10.1007/s44402-026-00157-6

**Published:** 2026-07-29

**Authors:** Pauline Kang, Gareth Lingham, Angelica Ly, Xianwen Shang, Regan Ashby, Serge Resnikoff, Fiona Stapleton, David Mackey

**Affiliations:** 1 School of Optometry and Vision Science, UNSW Sydney, Sydney, New South Wales Australia; 2 Centre for Ophthalmology and Visual Science (incorporating Lions Eye Institute), University of Western Australia, Perth, Western Australia Australia; 3 Centre for Eye Research Australia, Melbourne, Victoria Australia; 4 School of Optometry, The Hong Kong Polytechnic University, Hong Kong, China; 5 Faculty of Science and Technology, University of Canberra, Canberra, Australian Capital Territory Australia; 6 Brien Holden Vision Institute, Sydney, New South Wales Australia

**Keywords:** Adult myopia, Myopia, Prevalence, Systemic conditions

## Abstract

**Background:**

Data on myopia prevalence in Australia and associations with common systemic conditions and quality of life are limited. This study aimed to profile Australian adults self-reporting to have myopia, including co-morbidities and quality of life.

**Methods:**

This research included participants from the ongoing longitudinal 45 and Up Study. Sex and education data were collected at the baseline survey and additional data from the follow-up Health Compass survey. Responses on self-reported myopia, health and quality of life were analysed with logistic regression to assess the association between myopia and health conditions.

**Results:**

Of the 21,384 participant responses, 54.3% self-reported myopia, with higher rates in females and younger individuals. Self-reported myopes were more likely to have achieved higher levels of education, reside in urban and less remote regions and rate their quality of life and overall health worse than non-myopes. Education level was the strongest predictor of self-reported myopia. They were also more likely to report having visual or physical impairment, cardiovascular disease, chronic lung disease, diabetes and cancer. Non-myopic participants were less likely to report any disabilities or impairments and health conditions.

**Conclusion:**

The prevalence of self-reported myopia in Australian adults appears high and likely an overestimation. As commonly observed, self-reported myopia was higher in educated urban populations, with a greater propensity in females than males in the younger cohorts. Poorer quality of life and health ratings in self-reported myopes align with previous findings. The higher rates of health conditions in self-reported myopes may reflect shared lifestyle and environmental risk factors.

Key Points

- Self-reported prevalence of myopia in Australian adults was higher in females, younger individuals, urban residents and those with higher levels of education.
- Myopes were more likely to report having systemic conditions, including visual or physical impairment, cardiovascular disease, chronic lung disease, diabetes and cancer.
- Higher rates of health conditions in self-reported myopes may reflect common lifestyle and environmental risk factors.

## Introduction

The prevalence of myopia is rising globally [1, 2] and has reached epidemic levels in some parts of Asia [3, 4]. Given its association with sight-threatening ocular pathologies [5], myopia is one of the leading causes of irreversible visual impairment [6]. However, information on myopia in the Australian population is limited.

Mackey et al. [7] analysed data from several landmark Australian population studies to examine generational changes in refractive error among adults aged approximately 57–60 years from urban and rural populations. By comparing data from the early 1990s [8, 9] with more recent 2010s datasets [10, 11] for urban and rural adults, the authors found that myopia was more prevalent in Australians born after World War II than in earlier generations. This increase is likely due to higher education levels and reduced outdoor time, reflecting global increases in myopia across generations [7].

The prevalence of other chronic health conditions, such as cardio-metabolic diseases (e.g., diabetes and obesity) have also risen in Australia [12]. Like myopia, many of these conditions are believed to be driven by lifestyle changes associated with urbanisation, including dietary shifts and more sedentary behaviour [13]. Although the relationship between myopia and other chronic diseases remains underexplored, emerging evidence has suggested links with specific conditions such as insulin resistance and hyperinsulinemia [14, 15]. Insight into the association between myopia and acquired systemic conditions may enhance early detection and support holistic patient management, ultimately improving long-term health outcomes and reinforcing a shift toward a more holistic approach to myopia management that considers overall health and lifestyle factors alongside ocular outcomes. Thus, this exploratory study aimed to estimate the prevalence, distribution and characteristics of self-reported myopia among Australian adults aged 45 years and older using a longitudinal survey.

## Materials and Methods

### Study Design and Data Source

This research examined data obtained from the baseline and Health Compass follow-up surveys of the Sax Institute’s 45 and Up Study, Australia’s largest ongoing study of health and ageing. The 45 and Up Study is a large cohort longitudinal survey of persons aged 45 years and over and living in New South Wales (NSW), Australia [16, 17]. Participants were randomly selected from the Services Australia Medicare enrolment database. Individuals over 80 years of age and those living in rural and remote areas were oversampled [17]. The baseline survey asked questions relating to various health and social topics, including socio-demographic information, lifestyle choices and behaviours, quality of life and health conditions (https://www.saxinstitute.org.au/solutions/45-and-up-study/use-the-45-and-up-study/data-and-technical-information/). A total of 267,357 participants joined the 45 and Up Study by completing the baseline (Wave 1) survey between July 2005 and December 2009. The surveys were mailed to eligible participants and completed consent forms and questionnaires were returned via post; the response rate was 19% and included 11% of the NSW population aged 45 years and over.

All enrolled participants were invited to complete 5-yearly follow-up surveys [16] which included key questions designed to assess changes in socio-economic factors, health, lifestyle choice and behaviours. Additional themes were added to follow-up surveys to capture emerging issues likely to impact health, including vaccinations. The 45 and Up Health Compass survey was a recent follow-up survey conducted in July and August 2022, which aimed to understand the impact of recent changes in the organisation and delivery of healthcare, such as virtual healthcare and health monitoring devices. Participants were now 63 years and older when completing the Health Compass survey, and this could be completed either by post or online. A yes/no response question related to myopia was included, and 21,390 participants completed the survey question: ‘Have you ever needed to regularly wear glasses or contact lenses for short-sightedness (myopia)?’ Other questions in the 45 and Up study included in the analysis required responses in one of the following formats: multiple choice, binary response (yes/no), Likert scale or numerical response format (https://www.saxinstitute.org.au/solutions/45-and-up-study/use-the-45-and-up-study/data-and-technical-information/).

### Ethics and Consent to Participate and Publish

The overarching 45 and Up Study was approved by the University of New South Wales (UNSW) Human Research Ethics Committee (HREC) in 2005, and the 45 and Up Health Compass research project was approved by the UNSW HREC in 2022. The current study design and data reported here were also approved by the UNSW HREC (iRECS7321). Consent to participate and have their de-identified data published for approved studies was given by all participants in the 45 and Up Study.

### Data and Statistical Analysis

Self-reported data on gender and highest level of education were collected at the baseline survey (2005–2009) and were not reassessed in the subsequent Health Compass Survey (https://www.saxinstitute.org.au/solutions/45-and-up-study/use-the-45-and-up-study/data-and-technical-information/). All other data including body mass index (BMI), broad geography, needing correction for myopia, quality of life, overall health and self and family history of disabilities, impairments and health conditions were collected as part of the follow-up Health Compass survey in 2022 (https://www.saxinstitute.org.au/solutions/45-and-up-study/use-the-45-and-up-study/data-and-technical-information/). Secure data access was provided through the Sax Institute’s Secure Unified Research Environment (SURE).

Descriptive statistics determined frequencies and percentages for participant age, sex and BMI data. Broad geography data were used to categorise participants into rural versus urban locations based on Local Health District (geographically-based health administrative areas within NSW, Australia) and the Australian Bureau of Statistics Remoteness Area (RA) classifications (geographic remoteness across Australia based on access to essential services such as schools and hospitals) [18]. Six Local Health Districts cover metropolitan regions and are considered urban, while the remaining nine cover rural and regional NSW and are considered rural [19]. The five RA categories are: Major Cities, Inner Regional, Outer Regional, Remote and Very Remote.

Categorical variables were summarised using frequencies, percentages and numeric variables using median and interquartile range. Logistic regression was used to test for differences in the distribution of independent variables across levels of self-reported myopia, both before and after adjusting for age and sex. Likelihood ratio tests were used to assess whether a variable significantly improved the regression model fit. Analysis of deviance and likelihood ratio tests were used to assess whether a variable significantly improved the regression model fit. Given the descriptive nature of this study and its aim to understand the distribution of myopia and identify associated characteristics, not all analyses were adjusted for participant-level factors. Analyses were conducted through SURE in R version 4.4.1 (r-project.org), with a significance level set at *p* < 0.05.

## Results

### Demographic Information

A total of 21,390 participants completed both the 45 and Up Study baseline and the follow-up Health Compass surveys, and of those, 21,384 participants (42.8%, *n* = 9163 male) were available for analysis (mean [range] time between surveys of 14.4 [12.7–17.1] years). Of the 21,384 participants, 54.3% (*n* = 11,601) reported having regularly needed to wear some type of optical correction for myopia, and there was an overall effect of age (*p* < 0.001; Table 1), with the oldest age group having a lower reported need for optical correction for myopia than the youngest age group. The need for myopia correction was higher in females (56.7%) than in males (51.0%), with females having higher odds of self-reported myopia compared to males both before (OR, 1.25; 95% CI, 1.19–1.32; *p* < 0.001) and after adjusting for age group (OR, 1.24, 95% CI, 1.17–1.31, *p* < 0.001). The median BMI reported at the follow-up survey was comparable between participants with and without self-reported myopia (26.24 versus 26.10, respectively; *p* = 0.26).

**Table 1:** Number and percentages of participants in different age ranges and the highest level of education achieved (adjusting for age and sex), with and without self-reported myopia when completing the Health Compass survey, including the number of males (M) and females (F).

Ages				
**Age range (years)**	**No myopia**, ***n*** **(%)**	**Myopia**, ***n*** **(%)**	**Odds ratio**	***p* value**
55–64	2221 (45.1)	2703 (54.9)	Referent	-
	M = 871(50.6) F = 1350 (42.1)	M = 850 (49.4) F = 1853 (57.9)		
65–74	4813 (45.0)	5891 (55.0)	1.01 (0.94, 1.08)	0.87
	M = 2147 (47.9) F = 2666 (42.8)	M = 2333 (52.1) F = 3558 (57.2)		
75–84	2383 (46.6)	2727 (53.4)	0.94 (0.87, 1.02)	0.12
	M = 1241(48.2) F = 1142 (45.1)	M = 1336 (51.8) F = 1391 (54.9)		
85+	366 (56.7)	280 (43.3)	0.63 (0.53, 0.74)	**<0.001**
	M = 227 (59.0) F = 139 (53.3)	M = 158 (41.0) F = 122 (46.7)		

Highest level of education				
**Responses**	**No myopia**, ***n*** **(%)**	**Myopia**, ***n*** **(%)**	**Odds ratio**	***P* value**
No school certificate or other qualifications	214 (52.3)	195 (47.7)	Referent	-
	M = 97 (59.1) F = 117 (47.8)	M = 67 (40.9) F = 128 (52.2)		
School or intermediate certificate	1079 (49.4)	1106 (50.6)	1.08 (0.87, 1.33)	0.49
	M = 303 (55.7) F = 776 (47.3)	M = 241 (44.3) F = 865 (52.3)		
Higher school or leaving certificate^a^	794 (44.2)	1002 (55.8)	1.39 (1.12, 1.72)	**0.003**
	M = 393 (49.1) F = 401 (40.3)	M = 408 (50.9) F = 594 (59.7)		
Trade or apprenticeship	559 (52.8)	499 (47.2)	1.06 (0.84, 1.33)	0.64
	M = 443 (55.2) F = 116 (45.3)	M = 359 (44.8) F = 140 (54.7)		
Certificate or diploma^b^	2605 (48.2)	2796 (51.8)	1.17 (0.95, 1.43)	0.13
	M = 1110 (52.3) F = 1495 (45.6)	M = 1013 (47.7) F = 1783 (54.4)		
University degree or higher	4467 (42.9)	5954 (57.1)	1.47 (1.20, 1.79)	**<0.001**
	M = 2117 (45.2) F = 2350 (41.0)	M = 2569 (54.8) F = 3385 (59.0)		

Bold *p* values indicate statistical significance.

^a^Equivalent to secondary school level.

^b^Post-secondary school qualification below university degree level.

Education levels of participants with and without self-reported myopia, then further categorised by sex, are shown in Table 1. There was an overall effect of the highest level of education achieved before (*p* < 0.001) and after adjusting for age and sex (*p* < 0.001). Myopes were more likely to have achieved a higher level of educational attainment. Specifically, there was a significantly greater number of self-reported myopes with higher school or leaving certificates, as well as university qualifications, than non-myopes. Across all education levels, a higher proportion of females self-reported myopia than not. For males, a larger proportion self-reported not being myopic, except those who achieved higher school or leaving certificates and university qualifications (Table 2).

**Table 2:** Number and percentage of participants living in regions classified by Remoteness Area Level Categorisation, with and without self-reported myopia, along with the number of males (M) and females (F) within each region.

Local health district categorisation				
**Response**	**No myopia,** ***n*** **(%)**	**Myopia,** ***n*** **(%)**	**Odds ratio**	***p*** **values**
Urban	4307 (44.4)	5390 (55.6)	Referent	**-**
	M = 1969 (45.9) F = 2338 (43.2)	M = 2319 (54.1) F = 3071 (56.8)		
Rural	5406 (46.9)	6121 (53.1)	0.93 (0.88, 0.98)	0.006
	M = 2489 (51.6) F = 2917 (43.5)	M = 2335 (48.4) F = 3786 (56.5)		

Remoteness area level categorisation				
**Response**	**No myopia,** ***n*** **(%)**	**Myopia,** ***n*** **(%)**	**Odds ratio**	***p*** **values**
Major Cities of Australia	5560 (44.7)	6893 (55.4)	Referent	-
	M = 2452 (45.6) F = 3018 (43.2)	M = 2926 (54.4) F = 3967 (56.8)		
Inner Regional Australia	3406 (46.7)	3887 (53.3)	0.93 (0.88, 0.98)	**0.01**
	M = 1601 (52.8) F = 1805 (42.4)	M = 1434 (47.3) F = 2453 (57.6)		
Outer Regional Australia	772 (49.8)	779 (50.2)	0.83 (0.75, 0.92)	**<0.001**
	M = 423 (57.9) F = 448 (48.8)	M = 308 (42.1) F = 471 (51.2)		
Remote Australia	40 (52.0)	37 (48.1)	0.73 (0.46, 1.14)	0.17
	M = 17 (70.9) F = 23 (43.4)	M = 7 (29.2) F = 30 (56.6)		
Very Remote Australia^a^	5 (50.0)	5 (50.0)	0.88 (0.25, 3.19)	0.85
	M = <5 F = <5	M = <5 F = <5		

Bold *p* values indicate statistical significance.

^a^Data obfuscated due to risk of participant identification.

The number of participants with and without self-reported myopia from urban or rural/regional Local Health Districts are shown in Table 2. While a greater proportion of participants reported being myopic in both urban and rural regions, the prevalence of myopia was significantly higher in urban than rural regions before (*p* = 0.005) and after adjusting for age and sex (*p* < 0.001). In urban local health districts, both males and females showed a higher prevalence of myopia. In contrast, in rural Local Health Districts, a higher prevalence of myopia was observed only in females. Analysis by level of remoteness revealed a significant effect (*p* < 0.001), with participants living in inner and outer regional areas less likely to need correction for myopia (Table 2). Across all remoteness categories, a higher proportion of females than males reported being myopic, except in very remote regions.

Analysis of model deviance revealed that the level of education had the most significant impact on predicting whether a participant reported to be myopic or not, causing a change in deviance of 85.99 (43.97%). This was followed by sex (change in deviance of 66.86; 34.19%) and age group (change in deviance of 26.14; 13.37%), followed by the level of remoteness (change in deviance of 16.57; 8.47%)

### Health Outcomes

There were significant differences in the distribution of ratings of overall health (*p* < 0.001) but not quality of life (*p* = 0.06) between participants with self-reported myopia compared to those without (Table 3). Myopes were more likely to rate their overall health as fair or poor compared to non-myopes. Despite not reaching statistical significance, there was a similar trend for quality of life. When formally testing whether there was a trend between myopia prevalence and quality of life response (treating quality of life as an equally-spaced, ordered variable), each category decline in self-rated quality of life was associated with a 6% increase in the odds of self-reported myopia (OR = 1.06 [1.03,1.10]; *p* < 0.001), after adjusting for age and sex.

**Table 3:** Number and percentages of responses for each rating of quality of life and overall health rating after adjusting for age and sex, and rating of difficulty seeing, even with glasses, from participants with and without self-reported myopia.

Rate the quality of life						
**Rating**	**No myopia**, ***n*** **(%)**		**Myopia**, ***n*** **(%)**	**Odds ratio**		***P* value**
Excellent	2342 (47.0)		2640 (53.0)	Referent		-
Very good	5182 (45.9)		6100 (54.1)	1.05 (0.98, 1.12)		0.15
Good	1841 (44.2)		2328 (55.8)	1.14 (1.05, 1.24)		**0.002**
Fair	381 (44.1)		482 (55.9)	1.15 (1.00, 1.34)		0.06
Poor	37 (42.0)		51 (58.0)	1.27 (0.83, 1.96)		0.28

Rate overall health						
**Rating**	**No myopia**, ***n*** **(%)**		**Myopia**, ***n*** **(%)**	**Odds ratio**		***P* value**
Excellent	1171 (49.5)		1195 (50.5)	Referent		-
Very good	4691 (46.3)		5432 (53.7)	1.15 (1.05, 1.26)		**0.003**
Good	3008 (44.5)		3752 (55.5)	1.25 (1.14, 1.38)		**<0.001**
Fair	828 (43.1)		1092 (56.9)	1.34 (1.18, 1.51)		**<0.001**
Poor	85 (39.5)		130 (60.5)	1.54 (1.16, 2.06)		**0.003**

Difficulty seeing, even with glasses						
**Rating**	**No myopia**, ***n*** **(%)**	**Myopia**, ***n*** **(%)**			**Odds ratio**	***P* value**
No difficulty	7960 (48.0)	8607 (51.6)			Referent	-
Some difficulty	1787 (38.1)	2899 (61.9)			1.52 (1.42, 1.62)	**<0.001**
A lot of difficulty^a^	(27.5)	(72.5)			2.55 (1.72, 3.86)	**<0.001**
Cannot do at all^a^	(27.3)	(72.7)			2.42 (0.70, 11.1)	0.19

Bold *p* values indicate statistical significance.

^a^Data presented as percentages only due to the risk of participant identification.

Participants with self-reported myopia were more likely to report having some level of difficulty seeing compared to non-myopic individuals, with the odds ratio increasing with increasing level of difficulty (Table 3).

**Fig. 1:**
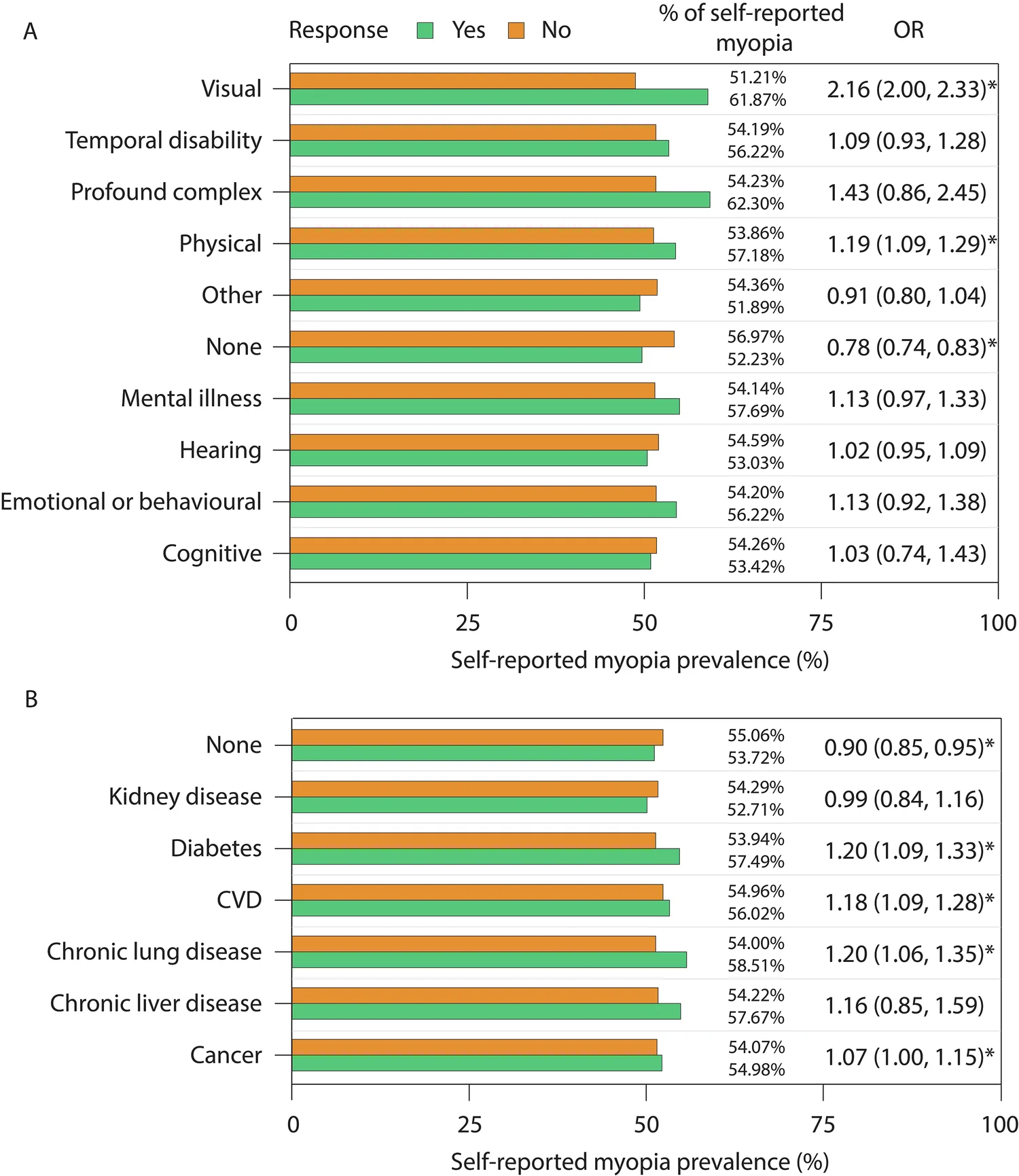
Participant responses to having **A** disabilities or impairments and **B** health condition(s), including the percentage of participants reporting to have myopia for both those with and without each condition, and odds ratio for a positive response from a participant with self-reported myopia. Asterisks indicate statistical significance of *p* < 0.05. CVD cardiovascular disease, OR odds ratio.

**Fig. 2:**
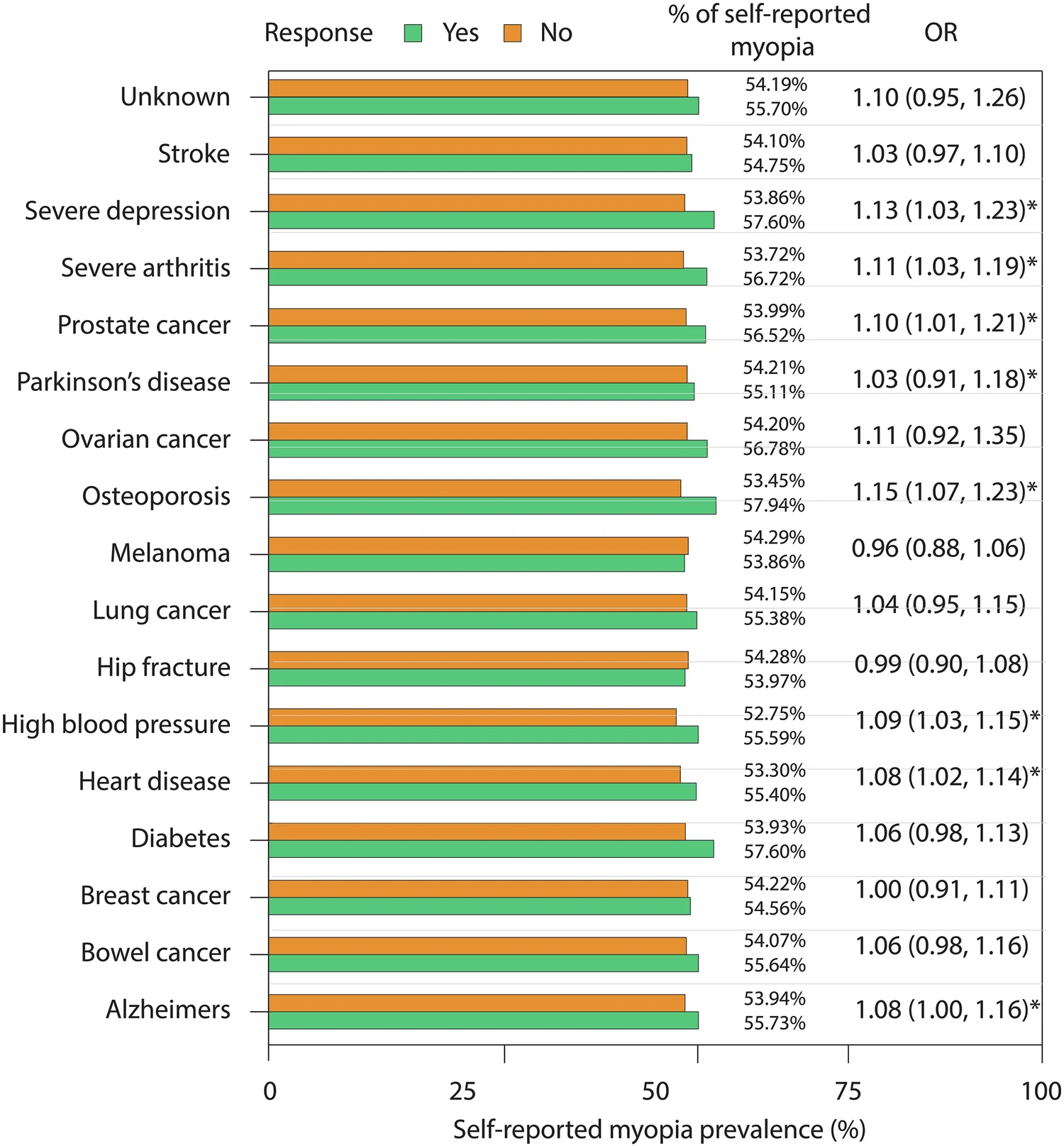
Participant responses to having a family history of health condition(s), including the percentage of participants reporting to have myopia for those with and without each condition, and the odds ratio for a positive response from a myopic participant. Asterisks indicate statistical significance of *p* < 0.05. OR odds ratio.

Figure 1 details responses regarding different types of disabilities or impairments and health condition(s) adjusted for age and sex. Participant family history (mother, father, brother or sister) of health conditions, also adjusted for age and sex, are shown in Fig. 2. Participants with self-reported myopia were more likely to report having visual or physical impairment, cardiovascular disease, chronic lung disease, diabetes and cancer compared to individuals without myopia. They were also less likely to report not having any visual or physical impairments compared to non-myopes. Additionally, myopes were more likely to have a family history of heart disease, high blood pressure, dementia/Alzheimer’s, severe depression, severe arthritis, prostate cancer and osteoporosis than non-myopes.

## Discussion

Emerging evidence suggests potential links between myopia and acquired systemic conditions, leading to growing interest in a more holistic approach to myopia management that considers broader health and lifestyle factors alongside ocular outcomes. Thus, this exploratory study aimed to estimate the prevalence, distribution and characteristics of self-reported myopia among Australian adults aged 45 years and older, to identify potential associations between participant factors.

Of the 21,384 participant responses, 54.3% reported having needed to wear a correction for myopia, with higher proportions of positive responses among females and younger age groups. This is greater than previously reported in Australian and other older adult populations and is likely an overestimation due to the self-reported nature of the survey. While myopia prevalence among children and adolescents varies widely across different regions, it tends to be less variable among older adults [20]. The regional Busselton Healthy Ageing Study (BHAS) and urban Gen 1 cohort of the Raine study, which included Australian adults of European descent with comparable age ranges to the current investigation, reported myopia prevalence of 19.6 and 29.9%, respectively. The European Eye Epidemiology (E^3^) Consortium found myopia prevalence rates of 30.6% in 25- to 89-year-old adults across Europe. Aligning with the current study, among those aged 55 years and older, myopia was more common in the younger than the older age groups, with no significant difference in prevalence between sexes [21]. In Singaporean adults aged 40–79 years, the prevalence of myopia was slightly higher at 38.7%. The distribution of myopia was bimodal, with higher prevalence in the 40–49 and 70–81 age groups compared to those in between, and increased myopia prevalence in the later years being attributed to lenticular changes [22]. Broader international population comparisons, such as those made by the Eye Diseases Prevalence Research Group, used data from six population-based studies of adults 40 years and older in the United States, Western Europe and Australia. Crude myopia (≤−1.00 D) prevalence in 2000 was estimated to be 25.4, 26.6 and 16.4% for adults in the United States, Western Europe and Australia, respectively [23]. Similar to reports from Singaporean adults, there was an increase in myopia in the oldest age group (≥80 years). While similar rates of myopia were noted between females and males, a higher prevalence of high myopia (≤−5.00 D) was observed in females [23].

When examining other factors influencing myopia prevalence, earlier studies of adult myopia often reported higher prevalence rates in males than females, whereas more recent evidence has identified the opposite trend [24]. Consistent with these contemporary findings, the current study found a higher proportion of females in the youngest age group (55–64 years) self-reporting myopia, while males in the same age group were equally likely to report being myopic or not. Education and lifestyle changes likely explain the generational changes in myopia prevalence between males and females [24]. A study of two Dutch cohorts from different generations found that older males often achieved higher educational levels, while boys from younger generations spent more time outdoors and less time reading compared to girls [24]. Adults who attain higher levels of education or spend more years in school tend to be more myopic, aligning with outcomes of the current study [25]. Interestingly, a higher proportion of females self-reported being myopic than not across all education levels. For males, this trend was only evident among those who had achieved higher school or leaving certificates and university qualifications. Of the factors examined, the analysis of deviance indicated that the highest educational attainment was a significant predictor of self-reported myopia, consistent with current evidence identifying education as a major risk factor for myopia [25].

Urban-rural disparities in myopia prevalence have been widely reported, with both children [26] and adults [27] from urban compared to rural regions being more likely to be myopic. Lifestyle, educational and population density differences are likely contributing factors. While the current data does not indicate where individuals lived when they developed myopia, the findings are consistent with the large body of work demonstrating higher prevalence in urban local health districts and major cities than in remote or rural regions.

When examining the impact of myopia on individuals, self-reported myopes in the current study reported poorer quality of life and overall health compared with non-myopes, and were more likely to select lower health ratings. Poorer quality of life is reported with uncorrected myopia, high myopia and those with complications of myopia [28]. While correcting the distance blur associated with myopia improves quality of life in children and adolescents, it does not restore it to a level experienced by an emmetrope [29]. In adults, myopia results in loss of productivity, with negative impacts on the quality of life [28]. Unfortunately, the current study was unable to determine the impact of myopia severity on overall health or quality of life ratings. These ratings were broad and general and did not account for multiple factors that may influence quality-of-life outcomes; accordingly, the results should be interpreted with caution.

Self-reported myopes were more likely to report having visual or physical impairment, cardiovascular disease, chronic lung disease and diabetes compared to individuals without myopia. The relationship between myopia and visual impairment identified in this study is unclear, as participants may have perceived myopia itself as a form of visual impairment. However, myopia negatively impacts physical functioning, which may correspond with participants’ understanding of physical impairment. In a study of individuals aged 22–76 years, most of whom were myopic (75%), participants reported that refractive error limited their ability to perform daily activities such as reading, driving and engaging in sports or leisure activities. Some also experienced difficulties with mobility [30].

Current research exploring links between myopia and systemic conditions remains limited. The observed increased likelihood of cardiovascular disease, chronic lung disease, cancer, diabetes and family history of health conditions among self-reported myopes observed in this study, compared to non-myopes, may be due to shared lifestyle and environmental risk factors. Sedentary behaviours, including watching television and occupational sitting, which are typically conducted indoors and are indicative of reduced time spent outdoors, have been linked with cardiovascular disease and certain cancers [31, 32]. Similarly, reduced time outdoors is an established risk factor for myopia [25]. Vitamin D levels have been used as a biomarker for time outdoors, with some studies finding reduced vitamin D serum levels in myopes compared to non-myopes [33]. Vitamin D deficiency has also been linked with various chronic lung diseases, and similarly, vitamin D supplementation can also reduce the risk of acute respiratory tract infections [34]. Air pollution, which is greater in urban compared to rural regions, adversely affects lung function and increases the risk of lung diseases and myopia, is similarly more prevalent in urban areas. Recent studies have also shown that exposure to air pollution increases the risk of myopia [35, 36]. Previous studies have postulated a link between abnormal ocular elongation, insulin resistance and hyperinsulinemia, which are key factors in the development of type 2 diabetes. Urbanisation leading to westernised diets and increased consumption of high glycaemic load food may change levels of growth factors and stimulate scleral tissue growth, resulting in axial elongation [14, 15]. Supporting this theory, a nationwide Israeli survey found that females with myopia during adolescence had a greater risk of type 2 diabetes in early adulthood, although this relationship was not found in males [37]. Myopia prevalence was also higher among young Danish adults with type 1 diabetes [38].

This study has several limitations. Refractive error was not measured, and it was assumed that participants accurately understood both the survey questions and the condition of myopia. It is possible that participants interpreted the question about needing to wear glasses or contact lenses for myopia as referring to any form of visual correction. Additionally, it is unclear whether myopia onset occurred during childhood or developed later in life from lenticular changes, which have been suggested to contribute to increased myopia prevalence in older adults [22, 23]. Information on health conditions was also self-reported, and there is the possibility of recall bias, which may affect data reliability. Additionally, certain groups, including myopes, may be more health-aware or more likely to report co-existing conditions, which may contribute to inflated associations and limit the ability to draw causal inferences. Baseline data on sex and the highest level of education were collected before other study information, as these variables were not reassessed in subsequent administrations of the Health Compass survey. The overall participation rate was low, with only 7% of participants who completed the initial baseline survey also completing the Health Compass follow-up survey. This may introduce a non-response bias where those with visual or health problems may have been more motivated to respond to the survey, although only 2% of questions were related to vision. The questionnaires were not validated, although most questions captured factual data rather than constructs which require validation. They were also completed via post or online, which can introduce some biases. Participants may have different access to post and online survey modes, and responses may vary depending on the mode by which the survey was completed, potentially introducing selection and measurement bias.

This exploratory study estimated the prevalence, distribution and characteristics of self-reported myopia among Australian adults who participated in the 45 and Up study. While 54.3% of participants reported needing to wear a correction for myopia, it is likely an overestimation due to the self-reported nature of the survey; the survey questions and condition of myopia may not have been understood accurately by participants. Myopia prevalence was also higher in younger age groups and females, and those reported to have myopia generally rated overall quality of life and overall health poorer than non-myopes. Study outcomes suggest a possible association between cardiovascular disease, chronic lung disease, diabetes and family history of health conditions and myopia, which may reflect shared lifestyle and environmental risk factors, warranting further investigation.

## Data Availability

The data that support the findings of this study are available from the Sax Institute; however, restrictions apply to the availability of these data and are therefore not publicly available. Data are available upon reasonable request and with permission of the Sax Institute.
